# Mechanism of Chinese Medicine Herbs Effects on Chronic Heart Failure Based on Metabolic Profiling

**DOI:** 10.3389/fphar.2017.00864

**Published:** 2017-11-22

**Authors:** Kuo Gao, Huihui Zhao, Jian Gao, Binyu Wen, Caixia Jia, Zhiyong Wang, Feilong Zhang, Jinping Wang, Hua Xie, Juan Wang, Wei Wang, Jianxin Chen

**Affiliations:** ^1^School of Preclinical Medicine, Beijing University of Chinese Medicine, Beijing, China; ^2^Beijing University of Chinese Medicine, Dongfang Hospital, Beijing, China; ^3^FengNing Chinese Medicine Hospital, FengNing, China

**Keywords:** chronic heart failure, UPLC-TOF/MS, syndrome, traditional Chinese medicine, metabolomics

## Abstract

Chronic heart failure (CHF) is a major public health problem in huge population worldwide. The detailed understanding of CHF mechanism is still limited. Zheng (syndrome) is the criterion of diagnosis and therapeutic in Traditional Chinese Medicine (TCM). Syndrome prediction may be a better approach for understanding of CHF mechanism basis and its treatment. The authors studied disturbed metabolic biomarkers to construct a predicting mode to assess the diagnostic value of different syndrome of CHF and explore the Chinese herbal medicine (CHM) efficacy on CHF patients. A cohort of 110 patients from 11 independent centers was studied and all patients were divided into 3 groups according to TCM syndrome differentiation: group of Qi deficiency syndrome, group of Qi deficiency and Blood stasis syndrome, and group of Qi deficiency and Blood stasis and Water retention syndrome. Plasma metabolomic profiles were determined by UPLC-TOF/MS and analyzed by multivariate statistics. About 6 representative metabolites were highly possible to be associated with CHF, 4, 7, and 5 metabolites with Qi deficiency syndrome, Qi deficiency and Blood stasis syndrome, and Qi deficiency and Blood stasis and Water retention syndrome (VIP > 1, *p* < 0.05). The diagnostic model was further constructed based on the metabolites to diagnose other CHF patients with satisfying sensitivity and specificity (sensitivity and specificity are 97.1 and 80.6% for CHF group vs. NH group; 97.1 and 80.0% for QD group vs. NH group; 97.1 and 79.5% for QB group vs. NH group; 97.1 and 88.9% for QBW group vs. NH group), validating the robustness of plasma metabolic profiling to diagnostic strategy. By comparison of the metabolic profiles, 9 biomarkers, 2-arachidonoylglycerophosphocholine, LysoPE 16:0, PS 21:0, LysoPE 20:4, LysoPE 18:0, linoleic acid, LysoPE 18:2, 4-hydroxybenzenesulfonic acid, and LysoPE 22:6, may be especially for the effect of CHM granules. A predicting model was attempted to construct and predict patient based on the related symptoms of CHF and the potential biomarkers regulated by CHM were explored.

This trial was registered with NCT01939236 (https://clinicaltrials.gov/).

## Introduction

Chronic heart failure (CHF), as a complex clinical syndrome, results from any structural or functional cardiac disorder that impairs the ability of the ventricle to fill with or eject blood (Hunt et al., 2005). In 2000, an international cooperation research program on cardiovascular disease in Asia (InterASIA) was made, which included 15,518 urban or rural residents from 35 to 74 years old. The results showed that the prevalence of heart failure was 0.9% for the general population, 0.7% for the males, and 1.0% for the females, indicating that there were ~4 million heart failure targets in China (Gu et al., 2003). CHF is the only cardiovascular disease with an increasing hospitalization burden and an ongoing drain on health care expenditures, for its complex molecular mechanisms cannot be easily deciphered (Ramani et al., 2010). As a complementary alternative, Traditional Chinese Medicine (TCM) may be to improve the state of CHF. Zheng (syndrome) is the key pathological principle of TCM. All diagnostic and therapeutic methods in TCM are based on the differentiations of syndrome, and this concept has been used for thousands of years in China (Gu, 1956; Li et al., 2007). Syndrome differentiation was based on information from traditional four diagnostic methods. For the complexity and multilevel relationships of four diagnostic methods, a mode which could distinguish syndrome differentiation and be verified is imperative. Many techniques of data mining are applied to syndromes in TCM (Lukman et al., 2007; Shi and Zhou, 2007; Gao et al., 2010; Yao et al., 2011). Chen et al. proposed a discovery algorithm based on revised mutual information to discover syndromes for chronic renal failure (Chen et al., 2007). In regards to coronary artery disease, Liu et al. designed standardization scale on inquiry diagnosis and constructed this diagnostic model by using the method of multi-label learning (Liu et al., 2010). With many achievements have been made in syndrome differentiation, there are still some problems left, deserving further discussion (Wang et al., 2009; Lu et al., 2012). Syndromes-identified techniques such as multi-label learning could identify syndrome information in TCM more effectively, and solve the multi-label problems of one sample with several syndromes. While there are many research challenges of multi-label learning to be addressed in the future, such as multi-label data usually suffers from inherent class imbalance and unequal misclassification costs. Metabolomics is distinctive for its overall concept and dynamic character, which may be an effective tool to reveal the scientific connotation of TCM. Metabolic alterations are measured in response to disease progression (Cheng et al., 2015). LC-MS based methods provide more compatible technique and high quality data for sensitive detection of small-molecule metabolites with robust reliability and reproducibility (Gika et al., 2007). Abnormal metabolism may characterize syndrome differentiation. Our previous metabolomics study on blood stasis syndrome of CHF showed glucose metabolism and lipid metabolism disorders reinforce each other, which results a deterioration of coronary artery disease with blood stasis syndrome. These metabolites maybe used as indicators of clinical diagnosis and treatment of coronary artery disease (Wang et al., 2011). Wang et al. concluded that NMR-based metabolomics approach demonstrated alteration of energy metabolism and other potential biological mechanisms underlying CHF (Wang et al., 2013b). In this paper, we try to discover comprehensive metabolomic characteristics of CHF and its syndrome differentiation for accurate clinical diagnosis. A syndrome predictive method for CHF was to build with non-targeted metabolomics and potential biomarkers related to CHF syndrome differentiation were to explore. The predictive performance would be validated and these potential biomarkers would be used as predictors to diagnose the patients. Besides, special metabolic characteristic of CMH on CHF would also be elaborated.

## Materials and methods

### Patients and study design

The study included 110 patients with chronic heart failure from 11 hospitals (The Second Affiliated Hospital of Beijing University of Chinese Medicine, Zhengzhou Hospital of TCM in Henan Province, The Affiliated Hospital to Changchun University of CM, Guang'anmen Hospital which was Affiliated to China Academy of Chinese Medicine Sciences, Hubei Provincial Hospital of TCM, Wuhan Hospital of TCM, Hospital of T.C.M.S Shijingshan District, Beijing Changping Hospital, Hospital of T.C.M.S Beijing Miyun County, Beijing Changping Hospital of Integrated Chinese and Western Medicine, TCM Hospital of Beijing Huairou and TCM Hospital of Tongzhou District) in China between May 2010 and December 2014. During the same period, 54 normal healthy participants of matched age were included from medical center of Dongzhimen Hospital as controls.

### Diagnostic criteria

Diagnostic criteria of CHF: 2007 China Guideline for the Diagnosis and Treatment of CHF (Chinese Society of Cardiology of Chinese Medical Association and Editorial Board of Chinese Journal of Cardiology, 2007). Heart function standard: The Criteria for Diagnosis and Treatment of Heart Disease first published by the New York Heart Association (NYHA) (Feinstein, 1964). Syndrome differentiation of CHF patients followed the TCM differentiation standard: according to the Guiding Principles for the Clinical Study of New Drugs in Traditional Chinese Medicine released in 2002 (Zheng, 2002).

### Inclusion criteria

Primary heart disease in this research was Coronary Heart Disease (CHD), which could be diagnosed by coronary computed tomography, coronary angiography, limb-salvage Q wave for electrocardiogram (ECG), history of acute myocardial infarction, ECG test, radionuclide examination support, etc. The included patients also had no history of taking antihypertensive drugs and exhibited a blood pressure under 160/100 mmHg or of hypertension. Following symptoms and signs were observed with a history of CHD: fatigue, difficulty breathing, fluid retention (edema), left ventricular enlargement, NYHA functional classification II or III, and end systolic volume of left ventricular increase and left ventricular ejection fraction (LVEF) < 40. All subjects between 40 and 75 years old; If there was a “No” answer for any issue, the subject could not enter into this research.

### Exclusion criteria

Patients with one of the following diseases were excluded: (1) serious valvular heart disease; (2) cardiomyopathy; (3) pericardial disease; (4) congenital heart disease; (5) cardiac shock; (6) acute myocardial infarction (AMI) within 4 weeks; (7) acute myocarditis or serious arrhythmia with variation in hemodynamics. Patients who suffer from pulmonary artery hypertension caused by cor pulmonale, pulmonary embolism, or stroke within a half year were also excluded. Patients who suffer from serious hepatic or renal deficiency. Patients who suffer from diseases of blood system or malignant tumor. Patients who suffer from diabetes mellitus with serious complications, hyperthyrea, or hypothyrea. Patients who suffer from infection. Pregnant women or women in lactation. Patients with mental disorders. Patients with infectious disease or who join in other trials within 2 months of the present study.

All qualified participants had signed informed consent prior to the enrollment. This study was conducted according to the principles of the Declaration of Helsinki and the principles of Good Clinical Practice.

All patients formed the chronic heart failure group, and were further divided into 3 groups according to TCM syndrome differentiation, including group of Qi deficiency syndrome (QD), group of Qi deficiency and Blood stasis syndrome (QB), and group of Qi deficiency and Blood stasis and Water retention syndrome (QBW). The normal healthy participants composed the NH group.

Besides, according to previous research (Wang et al., 2013a), the most syndrome of CHF was Qi deficiency and Blood stasis syndrome, so 64 of the participants with CHF of Qi deficiency and Blood stasis syndrome (QB group) were enrolled in a randomized double-blind controlled clinical trial. According to the 2007 China Guidelines for the Diagnosis and Treatment of Chronic Heart Failure (Chinese Society of Cardiology of Chinese Medical Association and Editorial Board of Chinese Journal of Cardiology, 2007), all patients in the two groups received the standardized western medicine treatment, which includes angiotensin-converting enzyme inhibitors (ACEI) or angiotensin receptor blockers (ARBs), b-blockers, and diuretics. Except the standardized western medicine treatment, subjects with the CHM treatment were defined as the CHM group, and subjects with the placebo intervention were defined as the placebo group. The subjects were treated with CHM granules, made from Astragalus membranaceus (Fisch.) Bge. var. mongholicus (Bge.) Hsiao (huangqi, 60 g), Codonopsis pilosula (Franch.) Nannf (dangshen, 15 g), Salvia miltiorrhiza Bge (danshen 15 g), Paeonia lactiflora Pall (chishao, 15 g), Prunus davidiana (Carr.) Franch. (taoren, 10 g), and Carthamus tinctorius L. (honghua, 10 g) or placebo granules twice per day for 4 weeks. The CHM granules and placebo granules were offered by Beijing Kang Rentang pharmaceutical industry (Beijing, China). Finally, 31 patients were in CHM group, and 33 were in placebo group.

Plasma samples were collected on morning of the first and the twenty-eighth day after enrollment and were immediately frozen at −80°C. Six minutes of walking test (6 MWT) and echocardiography were also detected at the same time. Both the observer of the 6 MWT and the echo technician were blind to the allocation of the patients.

After several preliminary experiments by different mixtures of methanol, acetonitrile, and methanol/acetonitrile (1:1), finally the extraction solvent was acetonitrile. By mixing equal amounts of plasma (30 μL) from all samples, pooled quality control samples were prepared to ensure data quality for metabolic profiling. Detailed sample methods were shown in the Supplementary Material.

This research was approved by the Institutional Committee on Human Research of Beijing University of Chinese Medicine (No. 200807007), and this trial is registered with NCT01939236 (Luo et al., 2015).

### Ultra performance liquid chromatography/time of flight mass spectrometry (UPLC-TOF MS) metabolomics study analysis

The liquid chromatographic separation of processed plasma was conducted on a 100^*^2.1-mm ACQUITY UPLC ° *R* BEH C18 1.7-um column (Lot No. 0252350221) using an ACQUITY Ultra Performance LC (Waters Corporation, Milford Massachusetts, USA), whereas mass spectrometry was performed on a SYNAPT G2 Quadrupole-Time of Flight system (Waters Corporation, Milford Massachusetts, USA). With a random-number generator in Excel (Microsoft, Redmond, Washington), 3 sequences for samples in discovery phase, validation phase and placebo group were assigned by the study administrator. During analyses of the sample sequence, after each 20 injections, then 1 quality control sample was run to check the stability of system. The acquired mass spectrometry data were exported to data format (.centroid) files by Masslynx Software (version 4.0, Waters Corporation). Data pre-treatment procedures, such as nonlinear retention time alignment, peak discrimination, filtering, alignment, matching, and identification, were performed in Progenesis QI (34 Maple Street, Milford Massachusetts, 01757, USA), and retention time and the mass-to ratio data pairs as the parameters for each ion. The UPLC-QTOF-MS characteristic chromatogram of CHM granules (6 herbs) was identified, and detailed method was shown in the Supplementary Material. Disturbed metabolites and metabolic pathways were identified by open database sources, including Human Metabolome Database (http://www.hmdb.ca/), METLIN (https://metlin.scripps.edu/), KEGG (http://www.genome.jp/kegg/pathway.html), and MetaboAnalyst (http://www.metaboanalyst.ca/faces/home.xhtml).

### Statistical analysis

By SIMCA (version 14.1, Umetrics AB, Umea, Sweden), the orthogonal projection to latent structure-discriminant analysis (OPLS-DA) model was applied to compare the differences in metabolic profiles between CHF, QD, QB, QBW, and NH group, and *P*-values, from the Student's *t*-test or Welch's *t*-test, depended on whether the variances of two populations are assumed to be equal by Levene test. The significant metabolic changes after treatments of Chinese Herb Medicine or Placebo were identified by Partial Least Squares Discriminant Analysis (PLS-DA) and OPLS-DA, and P values were from paired samples *t*-test. R2Y (cum) was used to estimate the “goodness of fit” of the model, and Q2(cum) to estimate the ability of prediction. All P values for each metabolite in 6 comparisons were corrected by Bonferroni correction and the false discovery rate (FDR) method for multiple testing across all metabolites within each comparison. Matplotlib version 2.0.2 and SciPy version 0.19.1 were used to conducted the heat maps and hierarchical cluster analyses. A standard linear Support Vector Machine (SVM) Classification (Bledsoe et al., 2016):

$$\begin{array}{l} f\left(x\right)=\text{sgn}\left[\left(w^{*}\cdot x\right)+b^{*}\right]=\text{sgn}\left\{\sum_{i=1}^{N}a_{i}^{*}y_{i}\left(x_{i}\cdot x\right)+b^{*}\right\},\;\\ \;\left(w^{*}x\right)+b\to 0\\ f\left(x\right)=\{\begin{array}{c} +1,\;x\in \omega_{1}\\ -1,\;x\in \omega_{2} \end{array} \end{array}$$

And detailed method was shown in the Supplementary Material. And receiver operating characteristic (ROC) analysis were used for diagnosis of different CHF syndromes and validation of CHM treatment effects. To correct potential overfit of 2 OPLS-DA comparisons of Prior & Post-CHM group and Prior & Post-Placebo group, five-fold Cross Validation was conducted to verify the specificity and sensitivity of the classifier. The data set was divided into 5 approximately equal bins and the models were evaluated 5 times. Statistical analyses were performed using python software version 2.7 (https://www.python.org/) with scikit-learn version 0.18.2 (http://scikit-learn.org). An adjusted *p* < 0.05 was considered statistically significant.

## Results

**Figure 1:**
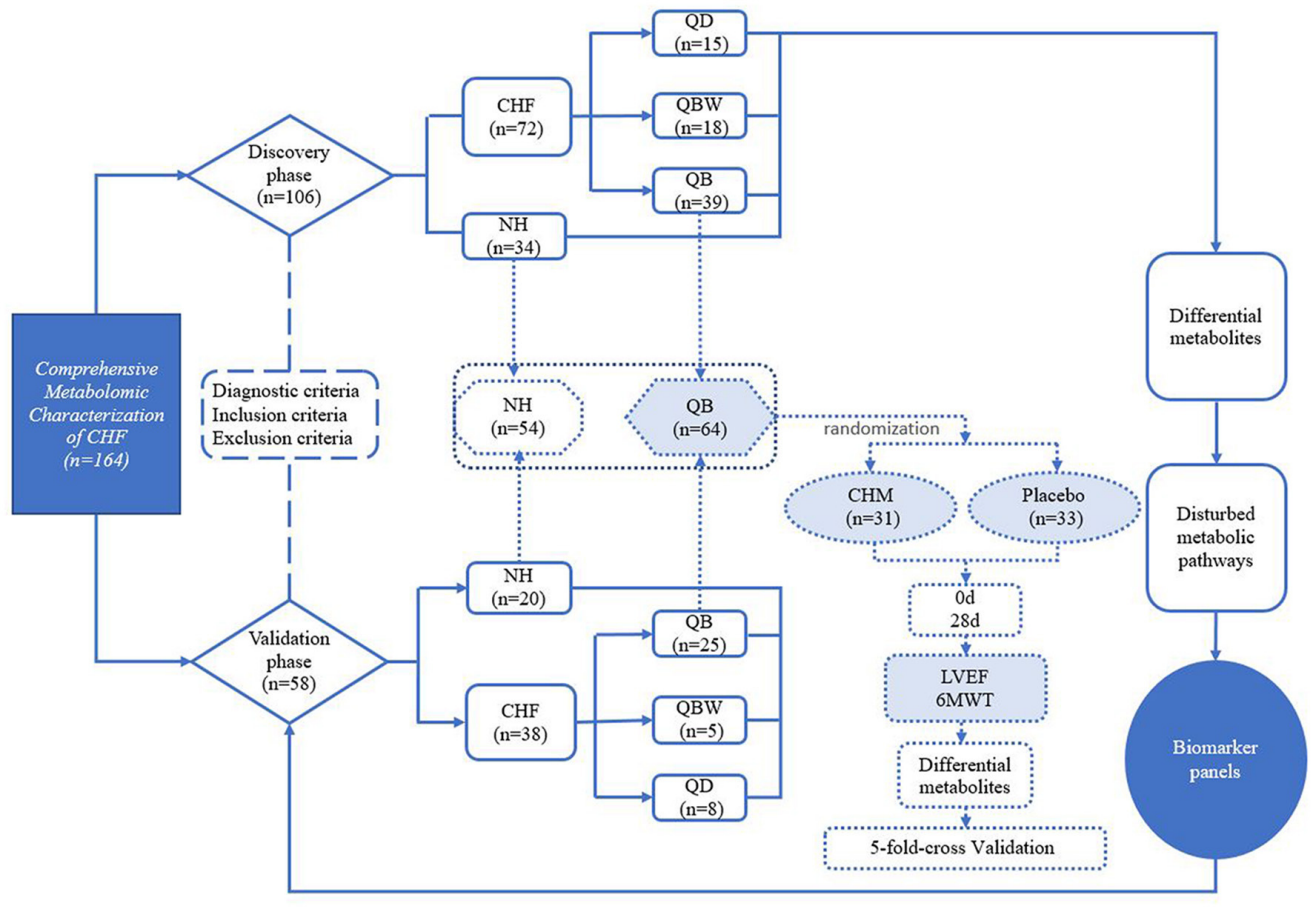
Study Design. 6MWT, 6 Min Walking Test; CHF, Chronic heart failure; CHM, Chinese herbal medicine; LVEF, Left Ventricular Ejection Fraction; NH, Normal healthy group; QD, Qi deficiency group; QB, Qi deficiency and Blood stasis group; QBW, Qi deficiency and Blood stasis and Water retention group.

A total of 110 patients with chronic heart failure from 11 hospitals and 54 normal healthy controls in China were enrolled in this research (Figure 1). The discovery phase included 34 normal healthy participants (NH) and 72 CHF participants, including the following groups: 15 QD, 39 QB, and 18 QBW patients. Table 1 showed the baseline characteristics and laboratory data. Compared with NH participants, LVEF and 6 MWT distance were lower in CHF patients. The CHF patients also had lower levels of alanine aminotransferase(ALT) and aspartate aminotransferase(AST), but higher levels of blood urea nitrogen(BUN), although there was no clinical significance.

**Table 1:** Baseline characteristics of discovery phase participants.

	**NH (*n* = 34)**	**QD (*n* = 15)**	**QB *(n* = 39)**	**QBW (*n* = 18)**	**CHF (*n* = 72)**	***P***
Age, yrs	66.47 ± 7.69	66.67 ± 8.01	70.03 ± 8.14	65.22 ± 7.86	68.13 ± 8.22	0.326
Female	55.9%	33.3%	51.3%	38.9%	44.4%	0.271
LVEF, %	59.26 ± 6.69	52.40 ± 13.70	49.03 ± 11.90	37.00 ±3.76	46.72 ± 12.24	0.000
6MWT	662.38 ± 44.47	328.92 ± 83.29	287.00 ± 102.33	221.27 ±118.77	277.71 ± 109.06	0.000
BMI, kg/m2	23.76 ± 3.86	26.00 ± 4.64	25.15 ± 3.12	23.58 ± 2.89	24.93 ± 3.49	0.120
**BLOOD PRESSURE, mm Hg**						
Systolic	134.50 ± 7.40	124.47 ± 6.55	128.08 ± 18.10	140.39 ± 13.47	130.40 ± 16.21	0.066
Diastolic	76.32 ± 8.35	75.80 ± 7.67	77.74 ± 12.49	85.00 ± 8.57	79.15 ± 11.17	0.192
Heart rate, beats/min	78.56 ± 9.34	76.27 ± 14.55	82.77 ± 21.74	86.22 ± 15.10	82.28 ± 19.00	0.784
**COMORBIDITY**						
Hypertension	61.80%	57.10%	75.70%	82.40%	73.50%	0.223
Diabetes mellitus	38.2%	35.7%	24.3%	50.0%	33.3%	0.624
Hyperlipemia	38.20%	50.00%	45.90%	5.90%	36.80%	0.885
Arrhythmia	17.60%	28.60%	37.80%	11.80%	29.40%	0.199
Smoking history	35.30%	33.30%	35.90%	44.40%	37.50%	0.826
Drinking history^*^	29.40%	40.00%	17.90%	22.20%	23.60%	0.522
**MEDICATION**						
ACEI/ARB	8.80%	60.00%	76.90%	88.90%	76.40%	0.000
Beta-blocker	14.70%	86.70%	76.90%	55.60%	73.60%	0.000
Antisterone	0.00%	33.30%	74.40%	44.40%	58.30%	0.000
Antiplatelet	14.70%	46.70%	61.50%	50.00%	55.60%	0.000
Statins	17.60%	80.00%	46.20%	38.90%	51.40%	0.001
Diuretic agent	0.00%	46.70%	82.10%	61.10%	69.40%	0.000
Digitalis	0.00%	13.30%	48.70%	27.80%	36.10%	0.000
**LABORATORY DATA**						
HGB	131.57 ± 33.15	140.13 ± 20.44	120.68 ± 39.07	131.09 ± 23.61	127.34 ± 33.10	0.540
ALT	31.22 ± 8.96	21.82 ± 14.50	22.27 ± 15.39	25.00 ± 15.82	22.87 ± 15.16	0.000
AST	28.07 ± 6.59	21.48 ± 7.47	23.20 ± 7.96	20.17 ± 7.99	22.07 ± 7.86	0.000
BUN, mmol/l	5.12 ± 1.60	6.18 ± 2.68	7.63 ± 4.07	6.92 ± 2.53	7.15 ± 3.49	0.002
CR, mmol/l	80.31 ± 15.53	71.95 ± 15.23	84.83 ± 31.88	84.68 ± 25.04	82.11 ± 27.70	0.612

*Values are mean ± SD or %*.

* *Patients with 50 ≥ g alcohol per day. P value for CHF vs. NH. 6MWT, 6 Min Walking Test; ACEI, angiotensin-converting enzyme inhibitor; ALT, alanine aminotransferase; ARB, angiotensin receptor blocker; AST, Aspartate transaminase; BMI, Body mass index; BUN, blood urea nitrogen; CHF, Chronic heart failure; CR, creatinine; HGB, hemoglobin; LVEF, left ventricular ejection fraction; NH, Normal healthy group; QB, Qi deficiency and Blood stasis; QBW, Qi deficiency and Blood stasis and Water retention; QD, Qi deficiency*.

The Validation phase included 20 NH participants and 38 CHF patients, of which baseline characteristics are shown in Supplementary Table 1.

### Comparisons of CHF groups & NH group and CHM group & placebo group

Representative based peak intensity (BPI) chromatograms of 4 samples with NH, QD, QB, and QBW, and 5 samples with NH, Prior & Post-CHM group, and Prior & Post-Placebo group obtained are shown in Supplementary Figures 1, 2. After peak alignment and removal of missing values, 442 negative-mode features were detected. After the OPLS-DA test, in SIMCA software, the ions with variable importance in the projection (VIP) values >1.0 and *p-*value <0.05 were considered the potential differential metabolites.

### Differential diagnosis of metabolic biomarkers in different syndromes

Presently clinical diagnosis of different TCM syndromes of CHF depends on personal experience of clinical doctors (Xu et al., 2016), so accurate diagnosis of the different stages of CHF, based on objective biomarkers in blood, is fundamental and significant for personalized treatment. So CHF and NH was compared to identify and characterize special metabolites of patients with CHF in discovery phase. QD, QB, and QBW were compared with NH to identify and characterize special metabolites of different Chinese syndromes in discovery phase.

**Figure 2:**
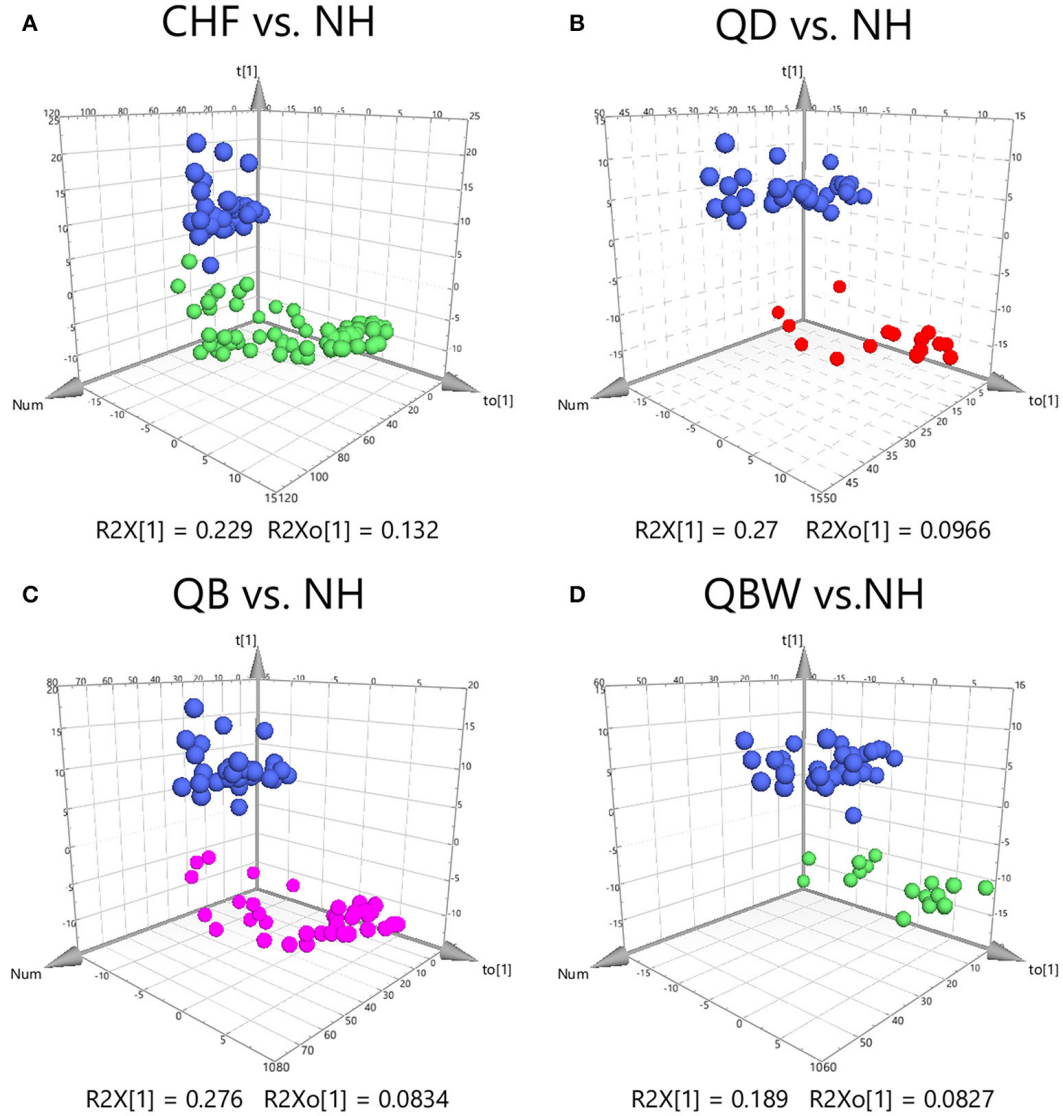
OPLS-DA Score Plots among CHF, QD, QB, QBW, and NH. OPLS-DA Score Plots compared **(A)** CHF vs. NH, **(B)** QD vs. NH, **(C)** QB vs. NH, and **(D)** QBW vs. NH. CHF, Chronic heart failure; NH, Normal healthy group; QD, Qi deficiency group; QB, Qi deficiency and Blood stasis group; QBW, Qi deficiency and Blood stasis and Water retention group.

Clear differences were obtained for the following: CHF vs. NH, cumulative R2Y at 0.923 and Q2 at 0.892 (Figure 2A); QD vs. NH, R2Y at 0.976 and Q2 at 0.94 (Figure 2B); QB vs. NH, R2Y at 0.967 and Q2 at 0.94 (Figure 2C); and QBW vs. NH, R2Y at 0.957 and Q2 at 0.868 (Figure 2D). With VIP values >1.0 and adjusted *p*-value < 0.05, the metabolites for each comparison appear in Table 2. Six specific metabolic biomarkers distinguished CHF from NH, 4 for QD from NH, 7 for QB from NH, and 5 for QBW from NH. PCA (Principal Component Analysis) result among QD, QB, QBW, and NH was shown in Supplementary Figure 3, and QD was the predominate Zheng contributing to CHF progression far away from NH.

**Table 2:** Statistical analysis of diagnostic biomarkers among NH and different CHF groups in discovery phase.

**Metabolites**	**Retention time (min)**	**Mass-to-charge ratio**	**VIP value**	Fold change^*^	***p-*Value^†^**	**Adjusted *p-*value^‡^**	**Adjusted *p*-value^§^**
**CHF vs. NH**							
LysoPC 15:0	15.44	480.3096	1.338	0.362	<0.0001	<0.001	<0.001
2-Arachidonoyl- glycerophosphocholine	15.43	540.3303	1.202	0.514	<0.0001	<0.001	<0.001
LysoPE 22:6	14.42	524.2772	1.191	0.274	<0.0001	<0.001	<0.001
PS 21:0	15.97	566.3457	1.114	0.566	<0.0001	<0.001	<0.001
Docosahexaenoic acid	19.07	327.2320	1.021	0.272	<0.0001	<0.001	<0.001
LysoPE 18:2	14.49	476.2777	1.002	0.542	<0.0001	<0.001	<0.001
**QD vs. NH**							
2-Arachidonoyl-glycerophosphocholine	15.43	540.3303	1.351	0.340	0.0012	0.005	0.005
PS 21:0	15.97	566.3457	1.194	0.338	0.0015	0.006	0.003
LysoPE 18:2	14.49	476.2777	1.115	0.244	<0.0001	<0.001	<0.001
LysoPE 20:4	14.51	500.2776	1.012	0.315	0.0001	<0.001	<0.001
**QB vs. NH**							
LysoPC 15:0	15.44	480.3096	1.449	0.200	<0.0001	<0.001	<0.001
2-Arachidonoyl-glycerophosphocholine	15.43	540.3303	1.343	0.358	<0.0001	<0.001	<0.001
LysoPE 22:6	14.42	524.2772	1.248	0.170	<0.0001	<0.001	<0.001
PS 21:0	15.97	566.3457	1.226	0.397	<0.0001	<0.001	<0.001
LysoPE 18:2	14.49	476.2777	1.082	0.405	0.0001	<0.001	<0.001
LysoPE 16:0	15.36	452.2775	1.031	0.459	0.0020	0.014	0.002
PS 19:0	13.97	538.3142	1.006	0.181	<0.0001	<0.001	<0.001
**QBW vs. NH**							
Uric acid	0.84	167.0212	1.665	0.282	<0.0001	<0.001	<0.001
LysoPE 18:0	17.64	480.3087	1.401	2.169	<0.0001	<0.001	<0.001
Indoxyl sulfate	1.62	212.0020	1.198	0.335	<0.0001	<0.001	<0.001
Arachidonic acid	19.41	303.2323	1.039	2.121	0.0043	0.021	0.005
LysoPE 16:0	15.36	452.2775	1.012	1.533	0.0005	0.003	<0.001

* *Fold change with a value>1 indicates a relatively higher concentration present in the CHF patients in 4 comparisons with NH*.

† *Student t test*.

‡ *Adjusted by Bonferroni correction across multiple comparisons within each metabolite*.

§ *Adjusted by false discovery rate method within each comparison*.

*CHF, Chronic heart failure; LysoPC, lysophosphatidylcholine; LysoPE, lysophosphatidylethanolamine; NH, Normal healthy group; PS, phosphatidylserine; QB, Qi deficiency and Blood stasis; QBW, Qi deficiency and Blood stasis and Water retention; QD, Qi deficiency; VIP, variable importance in the projection*.

**Figure 3:**
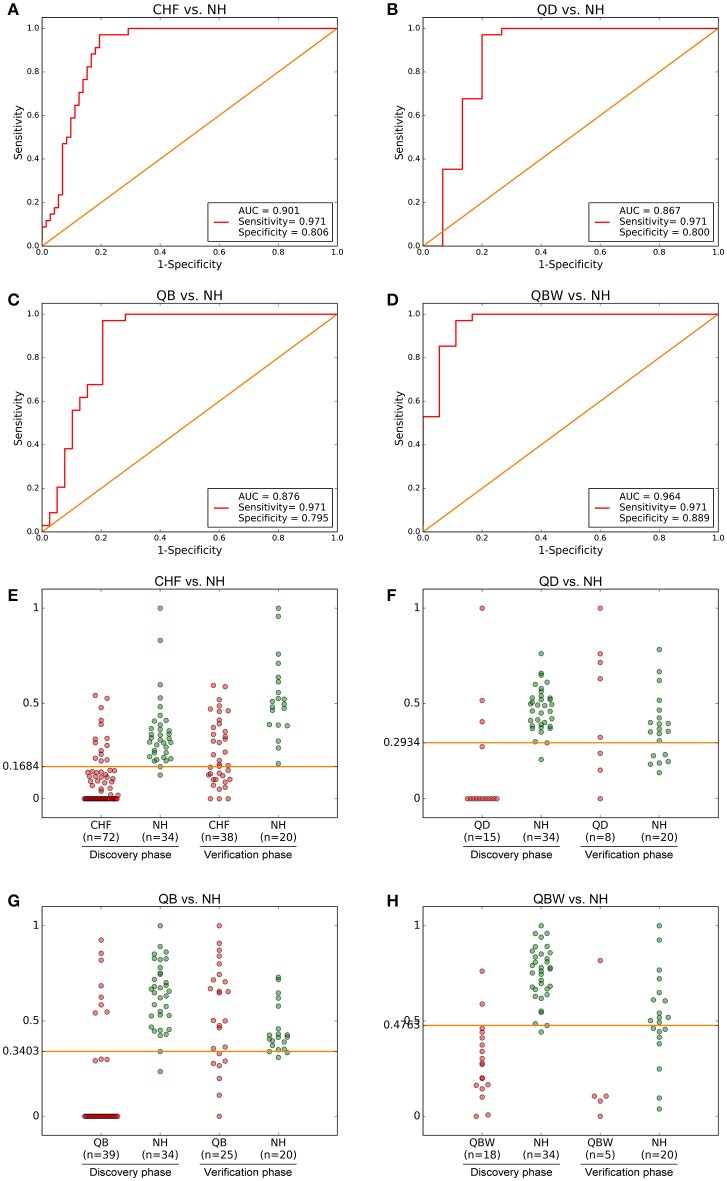
Diagnostic Outcomes and Predictive Accuracies. The diagnostic outcomes in the discovery phase are shown via the receiver-operating characteristic (ROC) curves for comparison between **(A)** CHF vs. NH, **(B)** QD vs. NH, **(C)** QB vs. NH, and **(D)** QBW vs. NH. The predictive accuracies by the biomarkers in validation phase and validation sets were compared between **(E)** CHF vs. NH, **(F)** QD vs. NH, **(G)** QB vs. NH, and **(H)** QBW vs. NH. AUC, Area Under the Curve; CHF, Chronic heart failure; NH, Normal healthy group; QD, Qi deficiency group; QB, Qi deficiency and Blood stasis group; QBW, Qi deficiency and Blood stasis and Water retention group.

The ROC presentations, on the basis of Support Vector Classification (SVC) of Support Vector Machine (SVM) which trained by each biomarker panel from the discovery phase, appear in Figure 3; the areas under the curve (AUC), sensitivity, and specificity are 0.901, 97.1%, and 80.6% for CHF vs. NH (*n* = 106; Figure 3A); 0.867, 97.1%, and 80.0% for QD vs. NH (*n* = 49) (Figure 3B); 0.876, 97.1%, and 79.5% for QB vs. NH (*n* = 73; Figure 3C); and 0.964, 97.1%, and 88.9% for QBW vs. NH (*n* = 52; Figure 3D), respectively.

With highest prediction specificity and sensitivity of the ROC, the optimal cut-off values were 0.1684 for CHF vs. NH, 0.2934 for QD vs. NH, 0.3403 for QB vs. NH, and 0.4763 for QBW vs. NH in the discovery phase. Different stages of CHF in the verification phase were predicted by the trained classifier and cut-off values. Predictive value was 85.0% for CHF vs. NH in the verification phase (Figure 3E); 95.1% for QD vs. NH in the verification phase (Figure 3F); for 99.1% QB vs. NH in the validation phase (Figure 3G); and 90.1% for QBW vs. NH in the validation phase (Figure 3H).

### Special metabolic treatment biomarkers of chinese medicine herbs

**Figure 4:**
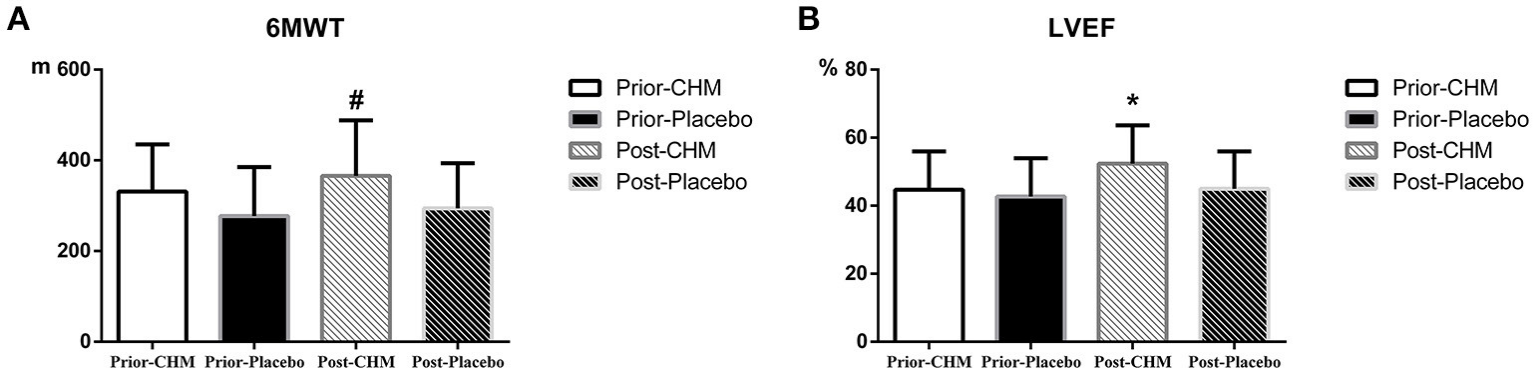
Result of 6MWT and ejection fraction results. **(A)** 6MWT result between Prior-CHM group and Prior-Placebo group & Post-CHM group and Post-Placebo group, ^#^*P* < 0.05 for Post-CHM group and Post-Placebo group. **(B)** Ejection fraction result between Prior-CHM group and Prior-Placebo group & Post-CHM group and Post-Placebo group, ^*^P < 0.05 for Post-CHM group and Post-Placebo group. 6MWT, 6 Min Walking Test; CHM, Chinese herbal medicine; LVEF, Left Ventricular Ejection Fraction.

There was no significant difference of 6 MWT and ejection fraction between Prior-CHM group and Prior-Placebo group, and 4 weeks later, both changed significantly between Post-CHM group and Post-Placebo group, as shown in Figure 4.

**Figure 5:**
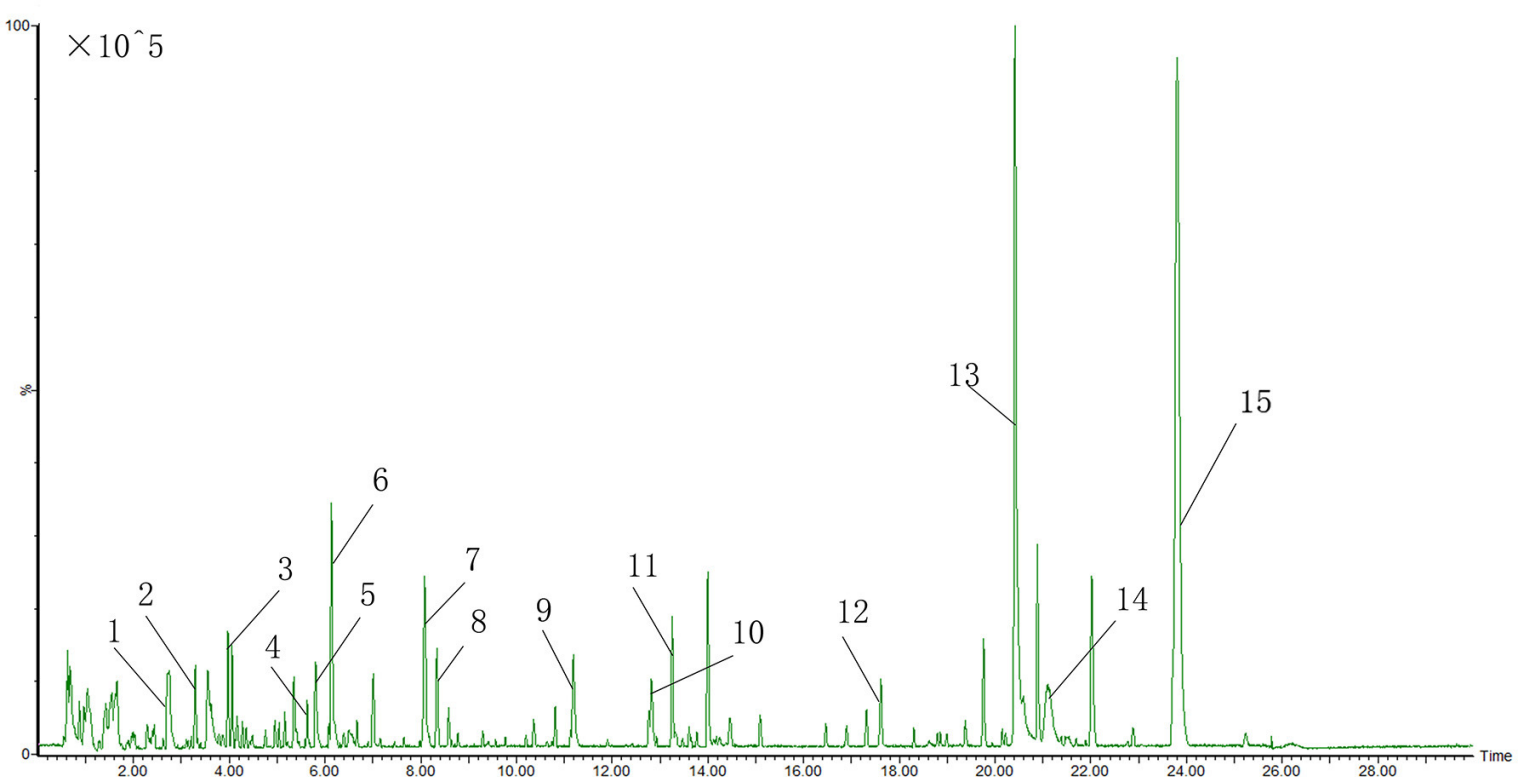
UPLC-QTOF-MS characteristic chromatogram of CHM granules (6 herbs). 1.Albiflorin; 2.Paeoniflorin; 3.Baicalin; 4.Ononin; 5.Tanshinol B; 6.Calycosin; 7.Formononetin; 8. Rhamnocitrin; 9.Dihydrotanshinone I; 10.Cryptotanshinone; 11.Dimethyl Lithospermate; 12.Tanshindiol B; 13.3-Indole Carboxylic Acid Glucuronide; 14.Beta-Carotene; 15.2-Nitrophenyl Beta-D-Glucuronide.

The UPLC-QTOF-MS characteristic chromatogram of CHM granules (6 herbs) was shown in Figure 5.

**Figure 6:**
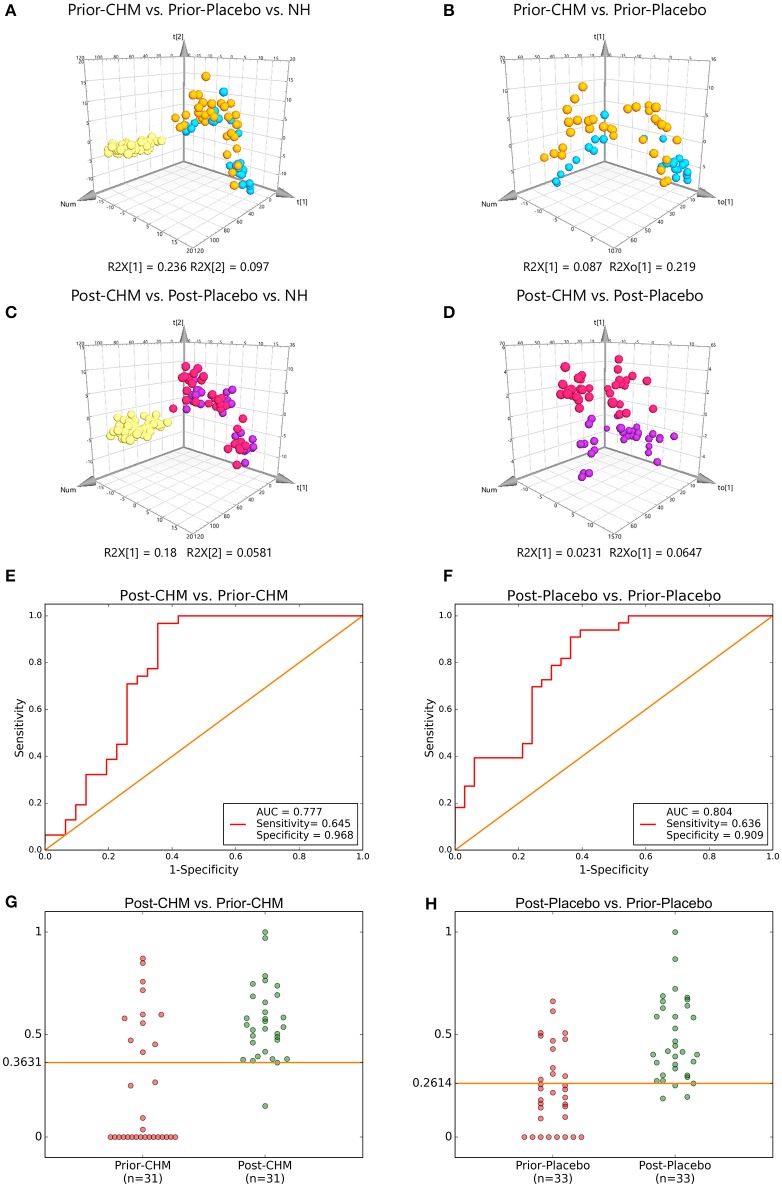
PLS-DA & OPLS-DA Score Plots and Treatment & five-fold Cross Validation Outcomes. PLS-DA Score Plots compared **(A)** Prior-CHM vs. Prior-Placebo vs. NH, **(C)** Post-CHM vs. Post-Placebo vs. NH. OPLS-DA Score Plots compared **(B)** Prior-CHM vs. Prior-Placebo, **(D)** Post-CHM vs. Post-Placebo. The treatment outcomes are shown via the receiver-operating characteristic (ROC) curves for comparison between **(E)** Post-CHM vs. Prior-CHM, **(F)** Post-Placebo vs. Prior- Placebo. The five-fold Cross Validation Outcomes were for **(G)** Post-CHM vs. Prior-CHM, **(H)** Post-Placebo vs. Prior-Placebo. AUC, Area Under the Curve; CHM, Chinese herbal medicine; NH, Normal healthy group. Green, NH; Yellow, Prior-Placebo; Blue, Prior-CHM; Red, Post-Placebo; Purple, Post-CHM.

There were significant curative effects of CHM on different diseases, but usually with indistinct metabolic mechanisms (Lao et al., 2014). According to 2 PLS-DA comparisons, Prior-CHM vs. Prior-Placebo vs. NH, R2Y at 0.586, Q2 at 0.524 (Figure 6A), Post-CHM vs. Post-Placebo vs. NH, R2H at 0.514, Q2 at 0.39 (Figure 6C), and 2 OPLS-DA comparisons, Prior-CHM vs. Prior-Placebo, R2H at 0.505, Q2 at 0.0866 (Figure 6B), Post-CHM vs. Post-Placebo, R2H at 0.845, Q2 at 0.16 (Figure 6D), after the 4 weeks treatment, the signs of patients in CHM group left the Placebo group to the NH group. Then Post-CHM group and Prior-CHM group was compared to identify and characterize special metabolites of combined treatment. Post-Placebo and Prior-Placebo was compared to identify and characterize special metabolites of basic Western Medicine treatment. The metabolites with VIP values >1.0 and adjusted *p*-values <0.05 for 2 OPLS-DA comparisons appear in Table 3 and there were 11 metabolic biomarkers for distinguishing Post & Prior-CHM group, and 3 for Post & Prior- Placebo group. By comparison between the 11 and 3 metabolic biomarkers, there were 9 special metabolic biomarkers for Prior & Post-CHM group.

**Table 3:** Statistical analysis of treatment biomarkers: post vs. prior-CHM and post vs. prior-Placebo.

**Metabolites**	**Retention time (min)**	**Mass-to-Charge Ratio**	**VIP value**	Fold Change^*^	***p*-Value^†^**	**Adjusted *p*-Value^‡^**	**Adjusted *p*-Value^§^**
**POST vs. PRIOR-CHM**							
2-Arachidonoyl-glycerophosphocholine	15.43	540.3303	1.818	2.389	<0.0001	<0.001	<0.001
LysoPC 15:0	15.44	480.3096	1.519	2.415	<0.0001	<0.001	<0.001
LysoPE 16:0	15.36	452.2775	1.482	2.994	<0.0001	<0.001	<0.001
PS 21:0	15.97	566.3457	1.477	2.087	0.0002	0.002	<0.001
LysoPE 20:4	14.51	500.2776	1.450	2.226	0.0002	0.002	<0.001
LysoPE 18:0	17.64	480.3087	1.423	2.456	0.0003	0.003	<0.001
Arachidonic acid	19.41	303.2323	1.348	2.627	0.0002	0.002	<0.001
Linoleic acid	19.67	279.2325	1.218	1.888	0.0053	0.059	0.007
LysoPE 18:2	14.49	476.2777	1.118	1.740	0.0305	0.336	0.037
4-Hydroxybenzenesulfonic acid	1.51	172.9915	1.109	2.224	0.0246	0.271	0.027
LysoPE 22:6	14.42	524.2772	1.049	2.183	0.0073	0.081	0.007
**POST vs. PRIOR-PLACEBO**							
Uric acid	0.84	167.0212	1.677	2.929	0.0009	0.003	0.003
LysoPC 15:0	15.44	480.3096	1.451	1.741	0.0010	0.003	0.002
Arachidonic acid	19.41	303.2323	1.215	1.736	0.0154	0.046	0.015

* *Fold change with a value>1 indicates a relatively higher concentration present*.

† *Student t test*.

‡ *Adjusted by Bonferroni correction across multiple comparisons within each metabolite*.

§ *Adjusted by false discovery rate method within each comparison. CHM, Chinese Herb Medicine; LysoPC, lysophosphatidylcholine; LysoPE, lysophosphatidylethanolamine; PS, phosphatidylserine; VIP, variable importance in the projection*.

The ROC presentations, on the basis of Support Vector Classification (SVC) of Support Vector Machine (SVM) which trained by on the basis of the logistic regression of each biomarker panel, appear in Figure 6; the AUC, sensitivity, and specificity are 0.777, 64.5%, and 96.8% for Post-CHM vs. Prior-CHM (*n* = 62; Figure 6E); and 0.804, 63.6%, and 90.9% for Post-Placebo vs. Prior- Placebo (*n* = 66; Figure 6F), respectively.

Then five-fold Cross Validation was conducted to verify the specificity and sensitivity of the classifier OPLS-DA results with Python packages “sklearn,” the result showed the optimal cut-off value was 0.3631, and true positive rate (TPR), false positive rate (FPR) were 96.7 ± 4.2, 19.2 ± 2.1% for Post-CHM vs. Prior-CHM (*n* = 62; Figure 6G); and 0.2614, 90.9 ± 1.2%, and 24.5 ± 2.2% for Post-Placebo vs. Prior-Placebo (*n* = 66; Figure 6H), respectively.

At the same time, there existed a certain correlation between specific metabolites determined in CHF patients with QB Zheng (*n* = 7) and CHM efficacy in QB patients (*n* = 11), evaluated by Chi-squared test with *P* = 0.0557.

### Correlation network of differential metabolites

**Figure 7:**
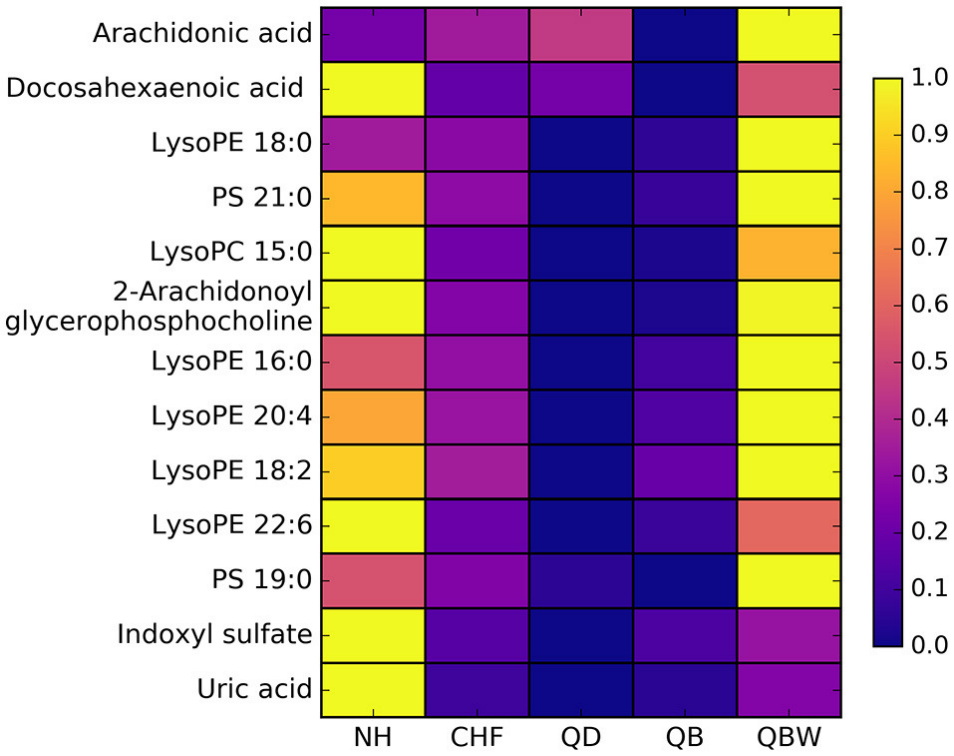
Heat map of differential metabolites. NH, Normal healthy group; QD, Qi deficiency group; QB, Qi deficiency and Blood stasis group; QBW, Qi deficiency and Blood stasis and Water retention group.

According to 4 OPLS-DA comparisons between Normal healthy group and different CHF groups, total 13 differential metabolites were identified. Average normalized quantities of the 13 differential metabolites were shown in the heat map Figure 7 among Normal healthy group and the CHF groups. Among them, 1 metabolites were elevated; 12 metabolites decreased in order according to the following groups: NH, CHF, QD, QB, and QBW.

**Figure 8:**
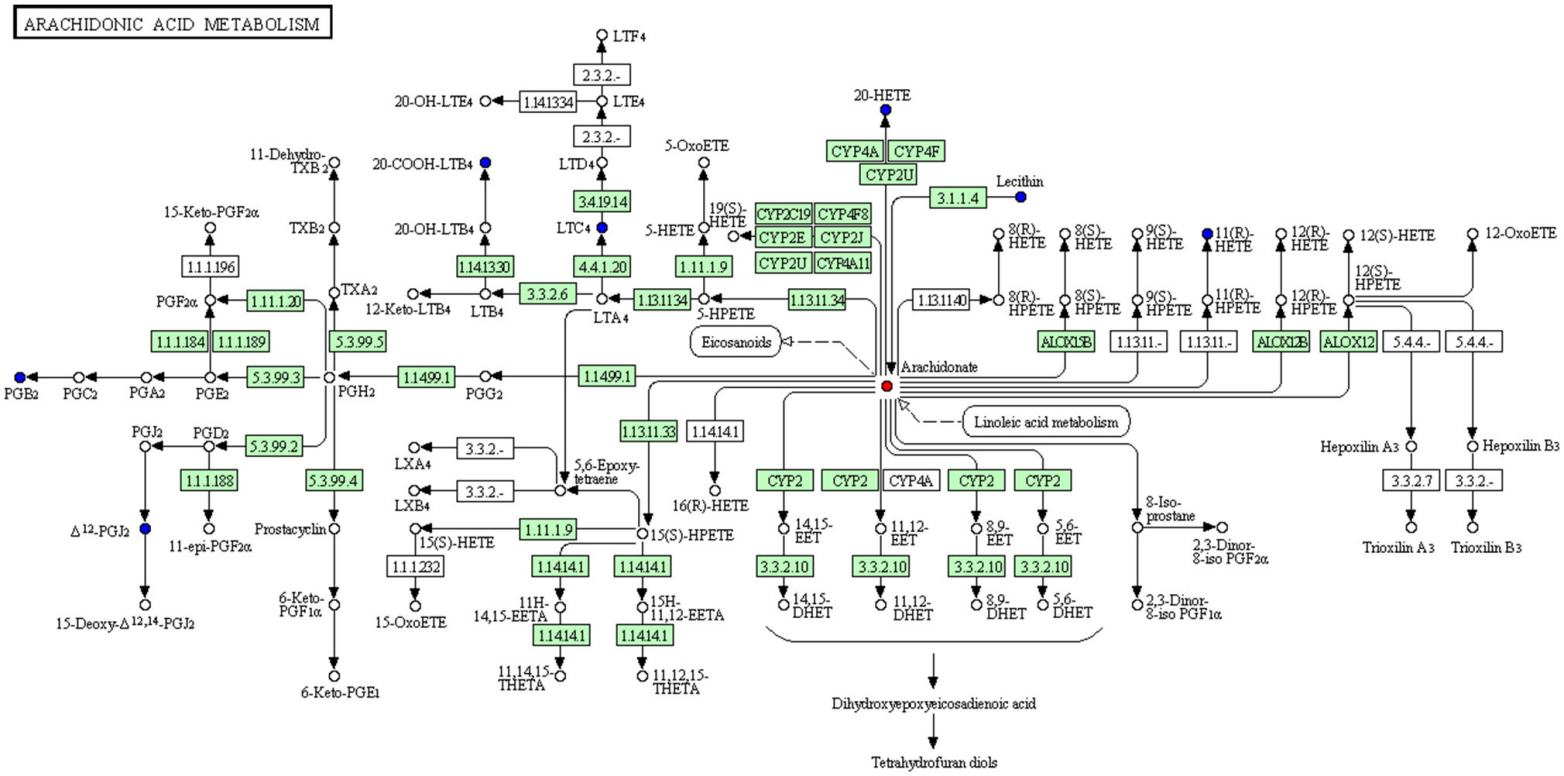
Metabolism-Protein Networks. Related proteins in Arachidonic acid metabolism pathway.

Moreover, we constructed the metabolism-protein networks and identify 36 related proteins, such as Calcium-dependent phospholipase A2, Group IIF secretory phospholipase A2, and Cytosolic phospholipase A2 in the most important Arachidonic acid metabolism pathway (Figure 8). Up-regulated and down-regulated metabolites determined were shown with red and blue points, and undetected metabolites are shown as gray points. Direct related enzymes were shown in green square frames, and others were with no color. Detailed information of points in Figure 8 could be found at http://www.kegg.jp/kegg/tool/conv_id.html.

The total 36 related proteins are shown in the Supplementary Material. The detailed information of Proteins and Pathways are shown in Supplementary Table 2.

## Discussion

Coronary artery disease is the leading cause of death in the world (Lu et al., 2012). In TCM, coronary artery disease belongs to the scope of chest heartache and cardiodynia (Liu et al., 2010). A comprehensive metabolomic evaluation in our research described for 110 patients who underwent CHF in 11 independent centers and 54 normal healthy participants in the discovery and validation phase. LC-MS-based metabolomic approach was conducted to demonstrate metabolic differences between NH and CHF patients with different syndromes in this study and evaluate the effect of Chinese medicine herbs on CHF.

Metabolite profiles analysis of plasma samples from CHF patients and CHF patients with different syndromes (Qi deficiency syndrome, Qi deficiency and Blood stasis syndrome, Qi deficiency and Blood stasis, and Water retention syndrome) could distinguish patients from normal healthy participants and provide fingerprints of metabolic changes that characterized the disease and its syndrome differentiation, and highlighted the potential biomarkers in the evaluation of a disease condition. About 6 metabolic biomarkers (LysoPC 15:0, glycerophosphocholine, LysoPE 22:6, PS 21:0, docosahexaenoic acid, and LysoPE 18:2) were highly possible to be associated with CHF, and 4 (2-arachidonoyl, PS 21:0, LysoPE 18:2, and LysoPE 20:4) with Qi deficiency syndrome, 7 (LysoPC 15:0, 2-arachidonoyl, LysoPE 22:6, LysoPE 18:2, LysoPE 16:0, PS 21:0, and PS 19:0) with Qi deficiency and Blood stasis syndrome and 5 (uric acid, LysoPE 18:0, indoxyl sulfate, arachidonic acid, and LysoPE 16:0) with Qi deficiency and Blood stasis and Water retention syndrome. The validated model using ROC curves was further constructed based on the metabolites of CHF patients and patients with 3 different syndromes and NH to diagnose other CHF patients with satisfying sensitivity of 97.1% (CHF), 97.1% (QD), 97.1% (QB), 97.1% (QBW), and specificity of 80.6% (CHF), 80.0% (QD), 79.5% (QB), 88.9% (QBW).

As the most common clinical CHF syndrome, patients with Qi deficiency and Blood stasis syndrome were enrolled for the evaluation of Chinese medicine herbs (CHM granules). In placebo group, there were 3 special metabolic biomarkers (uric acid, LysoPC 15:0, arachidonic acid) with the standardized western medicine treatment; in CHM group, there were 11 special metabolic biomarkers (2-Arachidonoylglycerophosphocholine, LysoPC 15:0, LysoPE 16:0, PS 21:0, LysoPE 20:4, LysoPE 18:0, arachidonic acid, linoleic acid, LysoPE 18:2, 4-hydroxybenzenesulfonic acid, and LysoPE 22:6) with the treatment of CHM granules and standardized western medicine. By comparison of the metabolic profiles, 9 biomarkers (2-arachidonoylglycerophosphocholine, LysoPE 16:0, PS 21:0, LysoPE 20:4, LysoPE 18:0, linoleic acid, LysoPE 18:2, 4-hydroxybenzenesulfonic acid, and LysoPE 22:6) may be especially for the effect of CHM granules.

In placebo and CHM groups, the levels of arachidonic acid both increased, while arachidonic acid is the potential biomarkers of Qi deficiency and Blood stasis and Water retention syndrome. As one of the pivotal signaling molecules, arachidonic acid is associated with inflammation, pain and self-balance function. Li et al. summarized the advances of giving insights into the arachidonic acid pathways in cardiovascular disease with metabolomic profiling (Ning et al., 2011). As a likely culprit for cardiovascular adverse effect associated with rofecoxib and NSAIDs, 20-hydroxyeicosatetraenoic acid (20-HETE) was identified during arachidonic acid metabolism in their study. On the side, it showed that epoxyeicosatrienoic acids (EETs) exhibited cardioprotective effects in a murine myocardial infarction model. It was reported that posphatidylcholine could produce proatherogenic species, choline, and trimethylamine oxide after metabolized by intestinal microbiota (Fan et al., 2016). It may be an implication for coronary artery disease with the decreasing level of phosphatidylcholine (Tang et al., 2013). Compared with patients in placebo group, patients in CHM groups had up-regulated more lysophosphatidyl ethanolamines. It was reported that the level of LysoPE 18:2 was down-regulated in patients with coronary atherosclerosis (Fan et al., 2016).

With the results of metabolites among each group, linoleic acid metabolism and arachidonic acid metabolism were identified as the main pathways according to the metabolism-protein network analysis and 63 potential related proteins included. Enzymes superfamily of Phospholipases A2 (PLA2s) are known to play multiple roles to maintain membrane phospholipid homeostasis and produce a variety of lipid mediators (Sun et al., 2010). It has been reported that increased or decreased expression of Ca2+-independent phospholipases A2 (iPLA2s) had profound effects on the metabolic state, CNS function, and cardiovascular performance, and dysregulation of iPLA2s can be a critical factor in the development of many diseases (Ramanadham et al., 2015). The superfamily of cytochrome p450 (CYP) takes part in the process of oxidation, peroxidation, and (or) reduction of vitamins, steroids, xenobiotics, and the majority of cardiovascular drugs in an oxygen- and NADPH-dependent manner. While during cardiovascular physiology and disease, the role of CYP is poorly understood. Evidence suggested that CYP plays an important role in the pathogenesis of cardiovascular diseases. The understanding was summarized as to the role that the receded CYP expression and (or) activity may play in the onset and progression of cardiovascular disease (Hunter et al., 2004).

## Conclusion

In this paper, a predicting model was attempted to construct and predict patient based on the related symptoms. The Syndrome prediction process was implemented by the objective biomarkers in blood forecasting performance. It generated a better performance than 3 classifiers with ROC curve analyses. We concluded that the method in the study may be applied to predict the syndromes of CHF. The potential biomarkers regulated by CHM were explored and it may be essential for the further study of CHM additional efficacy.

## Author contributions

KG, HZ, and JG: Designed the research, conducted performed the majority of the experiment, and revised the manuscript; BW, CJ, ZW, FZ, JiW, HX, and JuW: Assisted on supported several experimental performances and deal with the statistical data; JC and WW: Supervised the research and revised the manuscript.

### Conflict of interest statement

The authors declare that the research was conducted in the absence of any commercial or financial relationships that could be construed as a potential conflict of interest.

## Supplementary material

Supplementary Figure 1 Representative based peak intensity (BPI) chromatograms of samples with NH, QD, QB and QBW. NH, Normal healthy group; QD, Qi deficiency group; QB, Qi deficiency and Blood stasis group; QBW, Qi deficiency and Blood stasis and Water retention group.

Supplementary Figure 2 Representative based peak intensity (BPI) chromatograms of samples with NH, Prior & Post-CHM group and Prior & Post-Placebo group NH, Normal healthy group; CHM, Chinese herbal medicine group.

Supplementary Figure 3 PCA result among QD, QB, QBW and NH. 4NH, Normal healthy group; 1QD, Qi deficiency group; 2QB, Qi deficiency and Blood stasis group; 3QBW, Qi deficiency and Blood stasis and Water retention group.

Supplementary Table 1 Baseline Characteristics of Validation Phase Participants.

Supplementary Table 2 Proteins and KEGG Pathways list.

## Abbreviations

6MWT: 6 Minutes Walking Test
ACEI: Angiotensin-Converting Enzyme Inhibitor
ALT: Alanine Aminotransferase
ARB: Angiotensin Receptor Blocker
AST: Aspartate Transaminase
AUC: Areas Under the Curve
BMI: Body Mass Index
BUN: Blood Urea Nitrogen
CHD: Coronary Heart Disease
CHF: Chronic heart failure
CHM: Chinese herbal medicine
CR: Creatinine
ECG: electrocardiogram
FDR: false discovery rate
FPR: False Positive Rate
HGB: Hemoglobin
LVEF: Left Ventricular Ejection Fraction
LysoPC: lysophosphatidylcholine
LysoPE: lysophosphatidylethanolamine
NH: Normal healthy group
NYHA: New York Heart Association
OPLS-DA: Orthogonal Projection to Latent Structure-Discriminant Analysis
PCA: Principal Component Analysis
PLS-DA: Partial Least Squares Discriminant Analysis
PS: phosphatidylserine
QB: Qi deficiency and Blood stasis
QBW: Qi deficiency and Blood stasis and Water retention
QD: Qi deficiency
ROC: Receiver Operating Characteristic
SVC: Support Vector Classification
SVM: Support Vector Machine
TCM: Traditional Chinese medicine
TPR: True Positive Rate
VIP: Variable importance in the projection.

## References

[B1] Bledsoe J. C. Xiao D. Chaovalitwongse A. Mehta S. Grabowski T. J. Semrud-Clikeman M.. (2016). Diagnostic classification of ADHD versus control: support vector machine classification using brief neuropsychological assessment. J. Attent. Disord. [Epub ahead of print]. 10.1177/1087054716649666 27231214

[B2] Chen J. Guangcheng X. I. Chen J. Zhen Y. Xing Y. Wang J. (2007). An unsupervised pattern (Syndrome In Traditional Chinese Medicine) discovery algorithm based on association delineated by revised mutual information in chronic renal failure data. J. Biol. Syst. 15, 435–451. 10.1142/S0218339007002350

[B3] Cheng M. L. Wang C. H. Shiao M. S. Liu M. H. Huang Y. Y. Huang C. Y.. (2015). Metabolic disturbances identified in plasma are associated with outcomes in patients with heart failure: diagnostic and prognostic value of metabolomics. J. Am. Coll. Cardiol. 65:1509. 10.1016/j.jacc.2015.02.018 25881932

[B4] Chinese Society of Cardiology of Chinese Medical Association and Editorial Board of Chinese Journal of Cardiology (2007). Guidelines for the diagnosis and management of chronic heart failure. Chin. J. Cardiol. 35, 1076–1095. 10.3760/j.issn:0253-3758.2007.12.002 18341806

[B5] Fan Y. Li Y. Chen Y. Zhao Y. J. Liu L. W. Li J.. (2016). Comprehensive metabolomic characterization of coronary artery diseases. J. Am. Coll. Cardiol. 68:1281. 10.1016/j.jacc.2016.06.044 27634119

[B6] Feinstein A. R. (1964). Diseases of the heart and blood vessels: nomenclature and criteria for diagnosis. J. Am. Med. Assoc. 189, 869–870. 10.1001/jama.1964.03070110071036

[B7] Gao Z. Y. Zhang J. C. Xu H. Shi D. Z. Fu C. G. Qu D.. (2010). Analysis of relationships among syndrome, therapeutic treatment, and Chinese herbal medicine in patients with coronary artery disease based on complex networks. J. Chin. Integr. Med. 8:238. 10.3736/jcim20100307 20226145

[B8] Gika H. G. Theodoridis G. A. Wingate J. E. Wilson I. D. (2007). Within-Day reproducibility of an HPLC–MS-based method for metabonomic analysis: application to human urine. J. Proteome Res. 6, 3291–3303. 10.1021/pr070183p 17625818

[B9] Gu C. D. (1956). The Inner Classic of the Yellow Emperor, Essential Questions (Huangdi Neijing, Suwen). Beijing: People's Medical Publishing House.

[B10] Gu D. Huang G. He J. (2003). Investigation of prevalence and distributing feature of chronic heart failure in Chinese adult population. Chin. J. Cardiol. 31, 3–6.

[B11] Hunt S. A. Abraham W. T. Chin M. H. Feldman A. M. Francis G. S. Ganiats T. G.. (2005). ACC/AHA 2005 guideline update for the diagnosis and management of chronic heart failure in the adult: a report of the American college of cardiology/american heart association task force on practice guidelines (writing committee to update the 2001 Guideli. Circulation 112:e154. 10.1161/CIRCULATIONAHA.105.167587 16160202

[B12] Hunter A. L. Cruz R. P. Cheyne B. M. Mcmanus B. M. Granville D. J. (2004). Cytochrome p450 enzymes and cardiovascular disease. Can. J. Physiol. Pharmacol. 82, 1053–1060. 10.1139/y04-118 15644946

[B13] Lao Y. Wang X. Xu N. Zhang H. Xu H. (2014). Application of proteomics to determine the mechanism of action of traditional Chinese medicine remedies. J. Ethnopharmacol. 155:1. 10.1016/j.jep.2014.05.022 24862488

[B14] Li S. Zhang Z. Q. Wu L. J. Zhang X. G. Li Y. D. Wang Y. Y. (2007). Understanding ZHENG in traditional Chinese medicine in the context of neuro-endocrine-immune network. Iet Syst. Biol. 1, 51–60. 10.1049/iet-syb:20060032 17370429

[B15] Liu G. P. Li G. Z. Wang Y. L. Wang Y. Q. (2010). Modelling of inquiry diagnosis for coronary heart disease in traditional Chinese medicine by using multi-label learning. BMC Complement. Alternat. Med. 10:37. 10.1186/1472-6882-10-37 20642856 PMC2921356

[B16] Lu P. Chen J. Zhao H. Gao Y. Luo L. Zuo X.. (2012). *In silico* syndrome prediction for coronary artery disease in traditional chinese medicine. Evid. Complement. Alternat. Med. 2012:142584. 10.1155/2012/142584 22567030 PMC3328975

[B17] Lukman S. He Y. Hui S. C. (2007). Computational methods for Traditional Chinese Medicine: a survey. Comput. Methods Prog. Biomed. 88, 283–294. 10.1016/j.cmpb.2007.09.008 17983685

[B18] Luo L. Chen J. Guo S. Wang J. Gao K. Peng Z.. (2015). Chinese herbal medicine in the treatment of chronic heart failure: three-stage study protocol for a randomized controlled trial. Evid. Complement. Alternat. Med. 2015:927160. 10.1155/2015/927160 26089951 PMC4451157

[B19] Ning L. M. Liu J. Y. Hong Q. Harris T. R. Padmini Sirish M. S. Hammock B. D. (2011). Use of metabolomic profiling in the study of arachidonic acid metabolism in cardiovascular disease. Cong. Heart Fail. 17, 42–46. 10.1111/j.1751-7133.2010.00209.x PMC3583533 21272227

[B20] Ramanadham S. Ali T. Ashley J. W. Bone R. N. Hancock W. D. Lei X. (2015). Calcium-independent phospholipases A2 and their roles in biological processes and diseases. J. Lipid Res. 56:1643. 10.1194/jlr.R058701 26023050 PMC4548770

[B21] Ramani G. V. Uber P. A. Mehra M. R. (2010). Chronic heart failure: contemporary diagnosis and management. Mayo Clin. Proc. Mayo Clin. 85, 180–195. 10.4065/mcp.2009.0494 20118395 PMC2813829

[B22] Shi M. Zhou C. (2007). An approach to syndrome differentiation in traditional Chinese medicine based on neural network, in International Conference on Natural Computation (Haikou), 376–380. 10.1109/ICNC.2007.182

[B23] Sun G. Y. Shelat P. B. Jensen M. B. He Y. Sun A. Y. Simonyi A. (2010). Phospholipases A2 and inflammatory responses in the central nervous system. Neuromol. Med. 12, 133–148. 10.1007/s12017-009-8092-z 19855947 PMC3075861

[B24] Tang W. H. Wang Z. Levison B. S. (2013). Intestinal microbial metabolism of phosphatidylcholine and cardiovascular risk. N. Engl. J. Med. 368, 1575–1584. 10.1056/NEJMoa1109400 23614584 PMC3701945

[B25] Wang J. Chen C. Zhang P. Zhao H. H. Chen J. X. Luo L. T. (2013a). Distribution of TCM syndromes in 630 patients with chronic heart failures. J. Beijing Univ. Tradit. Chin. Med. 36, 567–571. 10.3969/j.issn.1006-2157.2013.08.016

[B26] Wang J. Li Z. Chen J. Zhao H. Luo L. Chen C.. (2013b). Metabolomic identification of diagnostic plasma biomarkers in humans with chronic heart failure. Mol. Biosyst. 9, 2618–2626. 10.1039/c3mb70227h 23959290

[B27] Wang Y. Li C. Guo S. Z. Chuo W. J. Chen J. X. Wang W. (2011). Serum metabolomics of blood-stasis syndrome of chronic myocardial ischemia based on CPMG pulse sequence. J. Beijing Univ. Tradit. Chin. Med. 34, 819–822.

[B28] Wang Y. Ma L. Liu P. (2009). Feature selection and syndrome prediction for liver cirrhosis in traditional Chinese medicine. Comput. Methods Prog. Biomed. 95, 249–257. 10.1016/j.cmpb.2009.03.004 19380172

[B29] Xu J. Xu Z. X. Lu P. Guo R. Yan H. X. Xu W. J.. (2016). Classifying syndromes in Chinese medicine using multi-label learning algorithm with relevant features for each label. Chin. J. Integr. Med. 22, 867–871. 10.1007/s11655-016-2264-0 27783322

[B30] Yao K. Zhang L. Wang J. Zhang J. (2011). Syndromes classification of the active stage of ankylosing spondylitis in traditional Chinese medicine by cluster analysis of symptoms and signs data. Commun. Comput. Inform. Sci. 86, 657–663. 10.1007/978-3-642-19853-3_97

[B31] Zheng X. Y. (2002). The Guiding Principles for the Clinical Study of New Drugs for Use In Traditional Chinese Medicine (In Trying). Beijing: Chinese Medical Science and Technology Press.

