# Supplementary figures and images for: Mechanism of Chinese Medicine Herbs Effects on Chronic Heart Failure Based on Metabolic Profiling
Source: Front Pharmacol. 2017 Nov 22;8:864. doi: 10.3389/fphar.2017.00864 (PMC5702651; doi:10.3389/fphar.2017.00864)

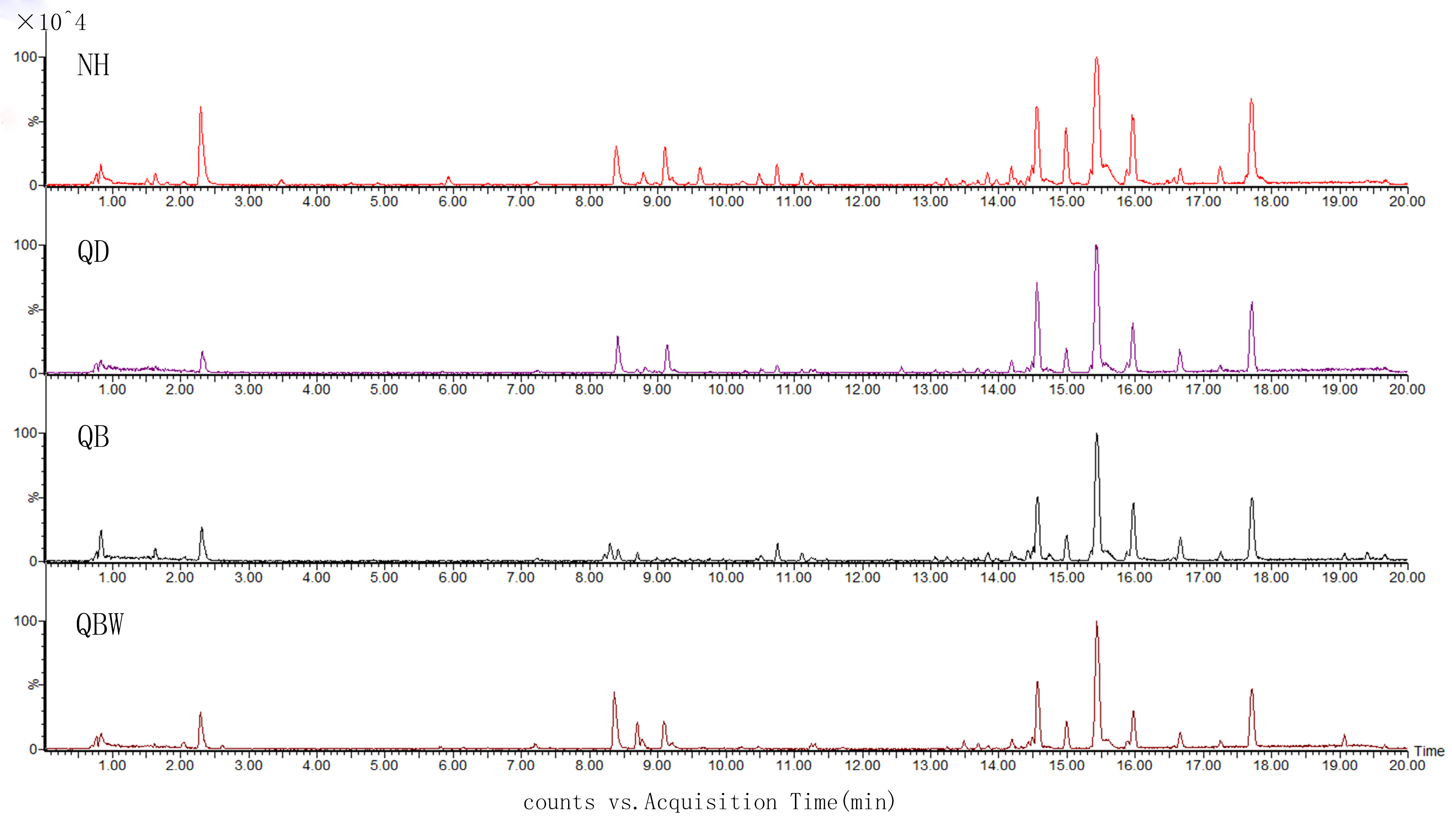

Supplement: Supplementary Figure 1 — Representative based peak intensity (BPI) chromatograms of samples with NH, QD, QB and QBW. NH, Normal healthy group; QD, Qi deficiency group; QB, Qi deficiency and Blood stasis group; QBW, Qi deficiency and Blood stasis and Water retention group. [file Image1.TIF]

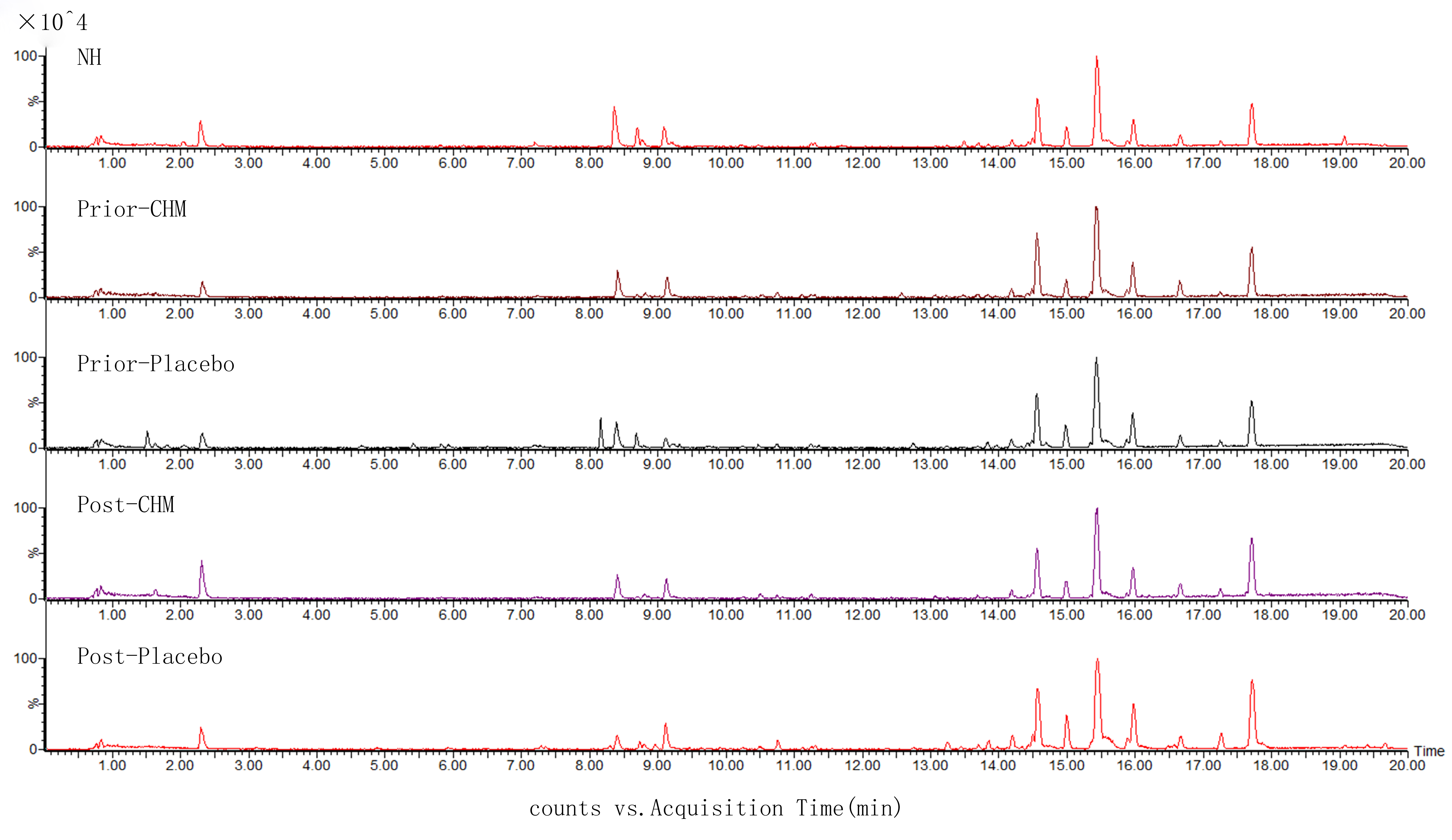

Supplement: Supplementary Figure 2 — Representative based peak intensity (BPI) chromatograms of samples with NH, Prior & Post-CHM group and Prior & Post-Placebo group NH, Normal healthy group; CHM, Chinese herbal medicine group. [file Image2.TIF]

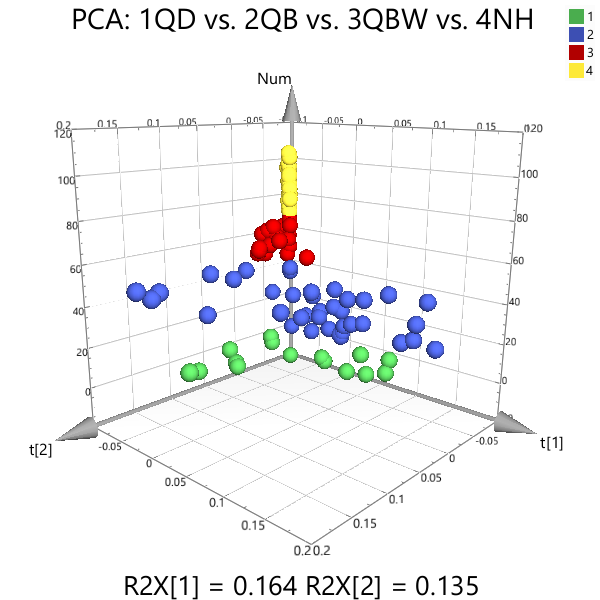

Supplement: Supplementary Figure 3 — PCA result among QD, QB, QBW and NH. 4NH, Normal healthy group; 1QD, Qi deficiency group; 2QB, Qi deficiency and Blood stasis group; 3QBW, Qi deficiency and Blood stasis and Water retention group. [file Image3.PNG]
