# Supplementary material for: Mechanism of Chinese Medicine Herbs Effects on Chronic Heart Failure Based on Metabolic Profiling
Source: Front Pharmacol. 2017 Nov 22;8:864. doi: 10.3389/fphar.2017.00864 (PMC5702651; doi:10.3389/fphar.2017.00864)
Supplement: Supplementary Table 1 — Baseline Characteristics of Validation Phase Participants. [file Table1.DOCX]

| ONLINE TABLE 1 Baseline Characteristics of Validation Phase Participants | | | | | | | |
| --- | --- | --- | --- | --- | --- | --- | --- |
|  |  | NH(n=20) | QD(n=8) | QB(n=25) | QBW(n=5) | CHF(n=58) | p |
| Age, yrs | | 67.99 ±6.60 | 65.38 ±8.28 | 68.76 ±6.15 | 69.60 ±8.56 | 68.16±6.90 | 0.719 |
| Female | | 25.00% | 25.00% | 44.00% | 0.00% | 34.20% | 0.471 |
| LVEF,% | | 62.67 ±5.35 | 35.50 ±5.50 | 36.12 ±5.41 | 57.60 ±12.78 | 38.82±9.87 | 0.000 |
| 6MWT | | 704.64 ±43.73 | 357.67 ±220.60 | 321.29 ±113.39 | 260.00 ±311.13 | 320.83±133.81 | 0.000 |
| BMI, kg/m2 | | 23.04 ±4.16 | 26.77 ±5.55 | 23.14 ±3.01 | 24.43 ±2.75 | 24.08±3.83 | 0.523 |
| Blood pressure, mm Hg | |  |  |  |  |  |  |
|  | Systolic | 118.56 ±9.85 | 134.13 ±17.62 | 128.32 ±14.52 | 128.60 ±15.96 | 129.58±15.12 | 0.010 |
|  | Diastolic | 72.64 ±8.11 | 78.50 ±13.37 | 78.88 ±8.28 | 73.60 ±12.03 | 78.11±9.86 | 0.038 |
| Heart rate, beats/min | | 71.24 ±7.98 | 80.63 ±13.66 | 78.12 ±12.72 | 76.00 ±14.70 | 78.37±12.86 | 0.069 |
| Comorbidity | |  |  |  |  |  |  |
|  | Hypertension | 55.00% | 100.00% | 84.20% | 75.00% | 85.70% | 0.018 |
|  | Diabetes mellitus | 40.00% | 66.70% | 41.70% | 0.00% | 36.80% | 0.839 |
|  | Hyperlipemia | 50.00% | 33.30% | 18.20% | 50.00% | 27.80% | 0.162 |
|  | Arrhythmia | 20.00% | 75.00% | 41.70% | 25.00% | 45.00% | 0.091 |
| Smoking history | | 55.00% | 50.00% | 54.20% | 80.00% | 56.80% | 0.898 |
| Drinking history* | | 30.00% | 25.00% | 32.00% | 60.00% | 34.20% | 0.745 |
| Medication | |  |  |  |  |  |  |
|  | ACEI/ARB | 5.00% | 75.00% | 96.00% | 60.00% | 86.80% | 0.000 |
|  | Beta-blocker | 15.00% | 75.00% | 88.00% | 60.00% | 81.60% | 0.000 |
|  | Antisterone | 0.00% | 12.50% | 36.00% | 60.00% | 34.20% | 0.002 |
|  | Antiplatelet | 15.00% | 62.50% | 76.00% | 40.00% | 68.40% | 0.000 |
|  | Statins | 20.00% | 12.50% | 52.00% | 40.00% | 42.10% | 0.092 |
|  | Diuretic agent | 0.00% | 25.00% | 68.00% | 60.00% | 57.90% | 0.000 |
|  | Digitalis | 0.00% | 25.00% | 32.00% | 40.00% | 31.60% | 0.005 |
| Laboratory data | |  |  |  |  |  |  |
|  | HGB | 131.73 ±4.64 | 127.96 ±21.32 | 131.22 ±13.84 | 122.00 ±32.38 | 129.32±18.27 | 0.386 |
|  | ALT | 26.68 ±5.08 | 23.50 ±10.45 | 20.92 ±12.01 | 13.05 ±9.35 | 20.63±11.53 | 0.002 |
|  | AST | 21.33 ±8.90 | 28.25 ±9.07 | 24.36 ±11.15 | 15.03 ±8.53 | 23.95±10.90 | 0.413 |
|  | BUN, mmol/l | 8.04 ±2.32 | 7.11 ±1.45 | 6.92 ±2.26 | 7.18 ±2.88 | 7.00±2.15 | 0.109 |
|  | CR, mmol/l | 54.97 ±15.23 | 93.00 ±54.88 | 78.45 ±22.37 | 102.52 ±32.24 | 84.68±33.03 | 0.000 |
|  |  |  |  |  |  |  |  |
| Values are mean±SD or %. *Patients with 50≥g alcohol per day. P value for CHF vs. NH.  6MWT=6 Minutes Walking Test; ACEI=angiotensin-converting enzyme inhibitor; ALT=alanine aminotransferase; ARB=angiotensin receptor blocker; AST=Aspartate transaminase; BMI=Body mass index; BUN=blood urea nitrogen; CHF=Chronic heart failure; CR=creatinine; HGB=hemoglobin; LVEF=left ventricular ejection fraction; NH=Normal healthy group; QB=Qi deficiency and Blood stasis; QBW=Qi deficiency and Blood stasis and Water retention; QD=Qi deficiency. | | | | | | | |
