# Supplementary material for: Mechanism of Chinese Medicine Herbs Effects on Chronic Heart Failure Based on Metabolic Profiling
Source: Front Pharmacol. 2017 Nov 22;8:864. doi: 10.3389/fphar.2017.00864 (PMC5702651; doi:10.3389/fphar.2017.00864)
Supplement: Supplementary file 6 [file DataSheet1.DOCX]

Online Supplementary

**Blood Sampling and Preprocessing**

5ml of fasting blood was drawn from each patient, by venous puncture on the elbow, on morning of the first and the twenty-eighth day after enrollment. The samples (collected in EDTA-K3, anticoagulant) were under 2800r/min centrifugation for 15 minutes at room temperature, and the supernatant was collected and then frozen and stored at -80 ℃ in the refrigerator for later test. After thawed at room temperature, 75μL of plasma was mixed with 300 μL of acetonitrile and the mixture was vortexed for 10 mins. Then, at 4°C, the mixture was centrifuged for 10 minutes at a rotation speed of 14,000 rpm. After that, 250 uL of supernatant was transferred to a clean tube and dried under a gentle stream of nitrogen at room temperature. Finally, the dried supernatant was dissolved with 100 μL water/acetonitrile (9:1) solution and 4 μL of the sample injected for UPLC-Q/TOF-MS analysis.

**UPLC-QTOF-MS characteristic chromatogram of the CHM granules (6 herbs)**

UPLC-grade acetonitrile is product of Fisher Scientific (Fair Lawn,NJ,USA). Formic acid is product of Sigma Aldrich (St.Louis,MO,USA). Ultrapure water is watsons water (Guangzhou).

*Sample preparation for analysis*

CHM granules (6 herbs) was weighed accurately(0.1g) and placed into a 1.5ml centrifuge tube containing 1.0ml methanol for 30 minutes sonicated. Following centrifugation at 14000rpm for 10 minutes in a centrifuge (Eppendorf, Melbourne, Australia). A supernatant (200ul) was transferred to the sample vial.

*UPLC-Q-TOF-MS conditions for chemical profile*

The UPLC analysis was carried out on a Waters UPLC (two LC-20ADXR solvent delivery units, a Binary solvent manager, sampler manager, and a SYNAPT G2 Q-TOF detector). The chromatographic separation was performed on a ACQUITY UPLC®BEH C18 column(2.1×100mm, 1.7μm) at 40℃. 0.1% aqueous formic acid (A) and acetonitrile containing 0.1% formic acid (B) were used as the mobile phase for analysis. The flow rate was set at 0.4mL/min. The elution condition was applied with a gradient program as follows: 0-20min, 5%B, 20-25min, 5-95%B, 25-25.01min, 95-5%B, 25.01-30min, 5%B. 2μL were injected into UPLC system for analysis.

**SVM Classification**

Given a training data set with $n$ samples, where each sample is associated with a set of p features a class label, represented by feature vector $X_{i}$ and binary class lable $Y_{i}$ in the form: $\left\{ x_{i}, y_{i} \right\}\in R^{p}\times\left\{ +1,-1 \right\},i=1,\ldots,n$. In this study, n is the number of participants in discovery phase, p is the number of statistically significant metabolites, and $y_{i}=1$ if participant $i$ is CHF, QD, QB or QBW and $y_{i}=0$ if participant $i$ is NH. Specifically, we have a training data set with $n=72,15,39,18$ for CHF, QD, QB and QBW $\left( y_{i}=1 \right)$ and 34 are NH$\left( y_{i}=0 \right)$. Each participant is associated with a number of statistically significant metabolites, which represent a feature vector of $p=6, 4, 7, 5$ features for CHF Vs. NH, QD Vs. NH, QB Vs. NH and QBW Vs. NH. A standard linear SVM algorithm fist maps all training samples $x$ into a $p$-dimensional space and then computs a decision function $f\left( x \right)=wx+b$ by adjusting weight of vector $\boldsymbol{W}:w_{j}\in R,j=1,\ldots,p$ to maximize the distance between samples $x_{i}$ and the linear boundary $\left( \boldsymbol{W},b \right)$ (aka hyperplane). Mathematically, victor $\boldsymbol{W}$ is orthogonal to the hyperplane, and $\frac{b}{\boldsymbol{W}}$ is the perpendicular distance from the hyperplane to the origin. The sign of $f\left( x \right)$ of sample $x$ indicates which side of hyperplane the sample resides and we can use this to represent its class label, As the objective of our classification study is to construct a simple classifier with a parsimonious decision model, an exhaustive forward feature selection technique was employed to select the smallest set of features to be included in the SVM classification. During training, the SVM model using only one features at a time and subsequently assing another feature if it improved the classification accuracy. It stopped when the accuracy satisfied a pre-specified threshold or reached a perfect classification.

**Detailed proteins in metabolism-protein networks**

Moreover, we constructed the metabolism-protein networks and identify 36 related proteins, including Calcium-dependent phospholipase A2, Group IIF secretory phospholipase A2, Cytosolic phospholipase A2, Phospholipase A2, Group XIIB secretory phospholipase A2-like protein, Group 10 secretory phospholipase A2, Group IIE secretory phospholipase A2, 85/88 kDa calcium-independent phospholipase A2, Group IID secretory phospholipase A2, Group 3 secretory phospholipase A2, Phospholipase B1 membrane-associated, Cytosolic phospholipase A2 beta, Group XIIA secretory phospholipase A2, Phospholipase A2 membrane associated, Cytochrome P450 4A11, Cytosolic phospholipase A2 gamma, Arachidonate 5-lipoxygenase, Arachidonate 15-lipoxygenase B, Arachidonate 12-lipoxygenase 12S-type, Prostaglandin G/H synthase 2, Prostaglandin G/H synthase 1, Arachidonate 15-lipoxygenase, Leukotriene-B(4) omega-hydroxylase 1, Leukotriene-B(4) omega-hydroxylase 2, Cytochrome P450 2C9, Cytochrome P450 2C19, Cytochrome P450 2E1, Cytochrome P450 2B6, Cytochrome P450 2C8, Cytochrome P450 4F8, Cytochrome P450 2J2, Arachidonate 12-lipoxygenase, 12R-type, Cytosolic phospholipase A2 delta, Cytosolic phospholipase A2 epsilon, Cytosolic phospholipase A2 zeta and HRAS-like suppressor 3, in main Arachidonic acid metabolism pathway.
